# Influence of repositioning guides’ color and usage on precision in tooth color measurement with a clinical spectrophotometer

**DOI:** 10.1590/1678-7757-2023-0348

**Published:** 2024-03-08

**Authors:** Tauan Rosa SANTANA, Paula Fernanda Damasceno SILVA, Márcia Luciana Carregosa SANTANA, Clara Lemos Leal Barata de MATTOS, André Luis FARIA-E-SILVA

**Affiliations:** 1 Universidade Federal de Sergipe Programa de Pós-graduação em Odontologia Aracaju SE Brasil Universidade Federal de Sergipe, Programa de Pós-graduação em Odontologia, Aracaju, SE, Brasil.

**Keywords:** Color, Data accuracy, Tooth discoloration, Tooth bleaching

## Abstract

Repositioning guides are commonly employed in clinical studies to ensure consistent tooth color measurements. Yet, their influence on measured color remains uncertain.

**Objective:**

This study evaluated the impact of repositioning guides’ color and usage on tooth color measurement using a clinical spectrophotometer.

**Methodology:**

In total, 18 volunteers participated in this study, in which the color of their upper left central incisor and upper left canine was measured with or without repositioning guides (control). The guides were made from pink, blue, or translucent silicone, as well as an acetate-based bleaching tray. Tooth color was measured in triplicates using a clinical spectrophotometer based on the CIELAB system. The standard deviations of these readings were used to estimate reproducibility, and color differences (ΔE00) between the measurements with guides and the control were calculated.

**Results:**

Repositioning guides had a minimal effect on L* values and no effect on b* values. The use of pink silicone increased a* values, whereas blue or translucent silicone reduced them. Irrespective of the evaluated tooth, the lowest ΔE00 values were observed for the translucent silicone and bleaching tray. The usage of guides only affected data variability for the L* color coordinate.

**Conclusion:**

Using repositioning guides can significantly impact the precision of tooth color measurement with a clinical spectrophotometer.

## Introduction

Tooth color measurement can be visually performed by selecting the tab from a shade guide that best matches the evaluated tooth.^1-3^ However, it is important to note that visual analysis may be affected by factors such as the operator’s experience, color discrimination ability, and lighting conditions, which can impact measurement reproducibility.^4-6^ To minimize operator dependency, instrumental methods are available. These methods include using clinical spectrophotometers, digital scanners, or a standardized photographic protocol combined with image-processing software to measure the color of a tooth.^7-10^ These instrumental approaches are often preferred in clinical studies due to their ability to provide more reliable and consistent results.

Clinical spectrophotometers are widely utilized in trials that involve tooth color evaluation due to their affordability and ease of use. In studies examining tooth bleaching protocols, it is crucial to repeat color measurements throughout the experiment to accurately calculate color changes. Ensuring the reproducibility of the method is vital in obtaining reliable results. However, teeth exhibit polychromatic properties, with colors becoming progressively darker toward cervical areas.^11,12^ Consequently, altering the position of the spectrophotometer tip during different readings can impact the calculation of color changes. To address this issue, repositioning guides have been employed to ensure consistent placement of the device tip, enabling the measurement of tooth color in the same area throughout the entire study.^13-17^Likely, it is expected that using repositioning guides results in less data variability than hand-free measurements when the color of a same tooth is assessed several times.

While repositioning guides are utilized to enhance measurement reproducibility, there is currently no standardized protocol for their construction. Different studies have reported variations in the materials and colors used for these guides.^13-17^ It is worth noting that the presence of a colored guide can potentially influence both the illumination of the teeth and the background color, suggesting that the guide color may impact color measurements.^18-20^ However, this aspect has received limited research attention. Therefore, the objective of this study was to assess the impact of using repositioning guides made from different colors of silicone or a bleaching tray on the color measurement of upper central incisors and canines employing a clinical spectrophotometer. We formulated two hypotheses for this study. We hypothesized that the color and translucency of the repositioning guide would fail to influence the measured tooth color or the variability of data when compared to the use of the spectrophotometer without any guide.

## Methodology

This study was nested in a randomized clinical trial that aimed to evaluate the impact of moistening the enamel before applying a 37% carbamide peroxide solution on the effectiveness of tooth bleaching. This study exclusively utilized tooth color data collected from a specific group of participants enrolled in the trial, specifically before undergoing any tooth bleaching procedures. The study protocol received approval from the scientific review committee and the committee for protecting human study participants at the Federal University of Sergipe (protocol CAAE 16843819.9.0000.5546) and was registered in the Brazilian Clinical Trials Registry (protocol number RBR-9gtr9sc). All participants provided their informed consent by signing a participation agreement for the study.

### Experimental design

The independent variables of this study were the tooth being analyzed (upper central incisor or upper canine) and the repositioning guide, which had five levels. The repositioning guides were made with silicone impression material (pink, blue, or translucent) or with a bleaching tray. The absence of a guide served as the control condition. The dependent variables included the color coordinates in the CIELAB system, the variability in data between the measurements of these coordinates, and the overall color difference (ΔE_00_) when compared to the color observed in the control condition.

### Sample size calculation

Considering the paired design for continuous outcomes, the calculation was performed for the F-test of repeated measures (RM) ANOVA within factors. The calculation was based on an effect size F of 0.25, a type I error of 0.05, a power test of 0.90, a single intervention, and 10 measurements (two teeth vs. five repositioning guides). The correlation among repeated measures was set at 0.5, and a spherical correction ε of 1.0 was applied. We utilized the statistical power analysis software G*Power 3.1.9.6 (developed by Franz Faul, University of Kiel, Germany). According to our calculations, a minimum of 17 participants were required to meet the predetermined parameters.

### Sample

We measured the color of upper right canines and upper right central incisors in a sample of 18 patients who were receiving treatment from the Restorative Dentistry discipline in the Department of Dentistry at the Dental School. The selected patients had no restorations or carious lesions on the evaluated teeth. Additionally, participants were excluded if they presented severe tooth discoloration (e.g., tetracycline stains), enamel hypoplasia, or fixed orthodontic appliances.

### Production of positioning guides

**Figure 1:**
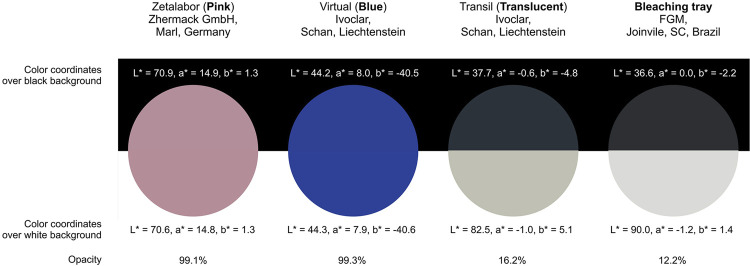
Illustrative images showing the color and opacity of silicone impression materials and the ethylene vinyl acetate bleaching tray used to obtain the repositioning guides. The circles corresponding to the different materials were colored using data from CIELAB coordinates measured over black and white backgrounds

To obtain stone working models, alginate (Hydrogum 5, Zhermack GmbH, Marl, Germany) was used to take impressions of participants’ upper arches. The repositioning guides were then produced on these molds using silicone-based impression materials, as described in Figure 1, which also provides information on the color and opacity of each material. All materials were mixed according to their manufacturer’s instructions. Specimens in the form of discs (10 mm in diameter, 3 mm in thickness) were made from each silicone mold to determine their optical properties. The color of these specimens was measured using a spectrophotometer (SP60, X-Rite, Grand Rapids, MI, USA) in reflectance mode against white (L* = 92.6; a* = 1.0, and b* = −0.5) and black (L* = 22.5, a* = 0.1, b* = 0.1) backgrounds. The opacity of the specimens was automatically calculated by the spectrophotometer, which was set to a 2° observer angle and a D65 illuminant with an aperture diameter of 8 mm. A bench spectrophotometer was utilized due to the inability of the clinical spectrophotometer to measure colors beyond the range of typical tooth shades.^21^

Repositioning guides were also constructed using customized bleaching trays made of ethylene vinyl acetate (Plates for Dental Trays Whiteness with 1 mm thickness, FGM, Joinvile, SC, Brazil). The color and opacity of a custom thermoforming tray (approximately 0.8-mm thick) were also measured and shown in Figure 1. The repositioning guides extended across both the buccal and palatal surfaces of participants’ teeth. A perforation with a diameter of 6 mm was created in the guides corresponding to the middle third of the buccal surfaces of the evaluated teeth. This diameter matched the tip of the spectrophotometer.

### Tooth color measurements

Before the color measurements, dental prophylaxis was performed using a rubber cup and pumice mixed with water. All color measurements were conducted using the clinical spectrophotometer VITA Easyshade V (Vita-Zahnfabrik, Bad Säckingen, Germany) in basic shade measurement mode. The device was regularly calibrated throughout the study. For each measurement, the brightness (L*), hue in the red-green axis (a*), and hue in the blue-yellow axis (b*) values were recorded.

The free-hand method without any repositioning guide served as the control condition. The spectrophotometer probe tip was placed in direct contact with the middle third of the teeth being evaluated. A single evaluator (T.R.S.) performed three measurements for each tooth. The same evaluator also assessed the color of the teeth in triplicates using the positioning guides. To determine data variability, standard deviation was calculated from the three measurements. Furthermore, Equation 1 was used to calculate the color difference between the values obtained using the positioning guides and those from the control:^22,23^


$$\text{ Equation 1: }\Delta E_{00}=\sqrt{{\left(\frac{\Delta L^{\prime }}{K_{L}S_{L}}\right)}^{2}+{\left(\frac{\Delta C^{\prime }}{K_{C}S_{C}}\right)}^{2}+{\left(\frac{\Delta H^{\prime }}{K_{H}S_{H}}\right)}^{2}+R_{T}\frac{\Delta C^{\prime }}{K_{C}S_{C}}\frac{\Delta H^{\prime }}{K_{H}S_{H}}}$$

Where ΔL’, ΔC’, and ΔH’ represent the changes in luminosity, chroma, and hue, respectively. SL, SC, and SH are the weighted functions for each component. KL, KC, and KH are the weighted factors for lightness, chroma, and hue, respectively (KL = KC = KH = 1). RT is the interactive term between chroma and hue differences.

### Data analysis

Before analysis, the normality of data distribution was assessed using the Shapiro-Wilk test (p>0.05 for all), and the homogeneity of variance was evaluated using Levene’s test (p>0.05 for all). The data for each color coordinate were then subjected to individual analysis using RM ANOVA, followed by the pairwise Tukey’s test. The factors examined were “tooth” and “repositioning guide,” both of which were considered as repetition factors. As for ΔE_00_, the data underwent RM ANOVA (tooth as repetition factor) and the Tukey’s test. A significance level of 95% was employed for all analyses.

## Results

### Measured color

The results for each color coordinate measured in relation to the factors “tooth” and “repositioning guide” are displayed in Table 1. The results of the RM ANOVA indicated that the factors “tooth” (p < 0.001) and “repositioning guide” (p < 0.001) had a significant impact on L* coordinate values. Furthermore, these factors had a significant interaction (p = 0.002). When examining the measurements of the upper central incisor, using pink or blue silicone as a guide resulted in the highest L* values, while the absence of a repositioning guide yielded the lowest values. Conversely, when assessing the color of the upper canines, the highest L* values were obtained without a repositioning guide or with a pink silicone guide.

**Table 1:** Means (standard errors) of color coordinates measured according to teeth and repositioning guide (n = 18)

Color coordinate		L*		a*		b*	
Tooth		Central incisor	Canine	Central incisor	Canine	Central incisor	Canine
Guides	None (freehand)	84.0 (0.9)^Ca^	82.0 (0.9)^ABa^	-1.27 (0.31)^Bb^	0.85 (0.29)^Ba^	18.4 (1.3)^ABb^	26.2 (1.5)^Aba^
	Pink silicone	88.2 (0.8)^Aa^	84.0 (1.0)^Ab^	0.24 (0.25)^Ab^	2.52 (0.24)^Aa^	19.0 (0.8)^Ab^	27.5 (1.3)^Aa^
	Blue silicone	86.7 (0.8)^Aa^	81.6 (1.2)^Bb^	-2.24 (0.27)^Cb^	-0.01 (0.39)^Ca^	16.4 (0.9)^Bb^	25.4 (1.7)^Ba^
	Translucent silicone	85.8 (0.8)^Ba^	82.0 (0.8)^ABb^	-1.82 (0.22)^Cb^	0.35 (0.37)^Ca^	17.2 (0.8)^Bb^	25.2 (1.7)^Ba^
	Bleaching tray	85.3 (0.9)^Ba^	82.0 (0.9)^ABa^	-1.55 (0.24) ^BCb^	0.71 (0.32)^BCa^	17.5 (0.8)^Abb^	26.2 (1.6)^ABa^

For each color coordinate, distinct letters (uppercase comparing the repositioning guides, lowercase comparing the teeth) indicate statistical difference at Tukey`s test (p < 0.05).

Regarding chromatic color coordinates (a* and b*), the interactions between the factors were insignificant (p = 0.870 for a* and p = 0.927 for b*). The factors “tooth” (p < 0.001) and “repositioning guide” (p < 0.001) had a significant effect on both coordinates. Regardless of the tooth being measured, using a pink silicone guide yielded the highest values for both a* and b*, whereas the blue and translucent silicones yielded the lowest values.

**Figure 2:**
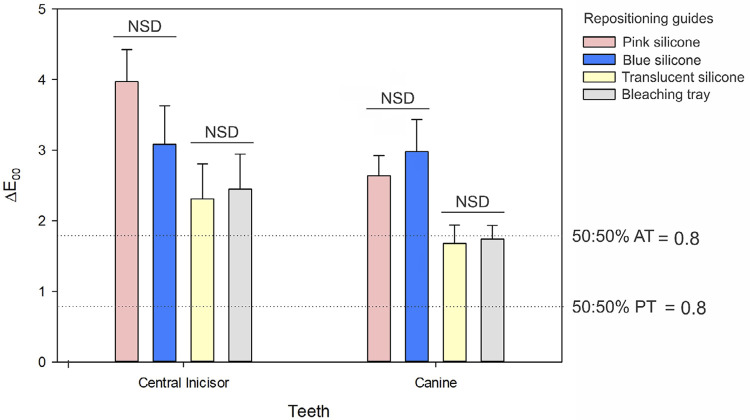
Means (standard errors) of ΔE00 calculated based on the color coordinates measured without any repositioning guide. NSD: non-significant difference. AT: acceptability threshold; PT: perceptibility threshold.^20^

Figure 2 displays the results for the overall differences (ΔE_00_) in color measurements when no repositioning guide was used. The RM ANOVA revealed that only the factor “repositioning guide” had a significant effect on ΔE_00_ values (p < 0.001), whereas the factor “tooth” (p = 0.178) and the interaction between the factors were insignificant (p = 0.085). Regardless of the tooth being measured, the highest color discrepancies from the control were observed when pink and blue silicones served as repositioning guides, with no significant differences between them. Similarly, utilizing either a translucent silicone guide or a bleaching tray resulted in similar ΔE_00_ values.

### Data variability

Table 2 presents the data variability of color measurements, indicated by the standard deviations for each coordinate. In terms of data variability between measurements for the same teeth, using a repositioning guide had a significant effect on L* values (p = 0.003). However, the factor “tooth” had no significant impact on lightness values (p = 0.053) and factors showed no significant interactions (p = 0.673). The highest variability in data was observed when no positioning guide was used, whereas the lowest, when either the pink or blue silicone guide was utilized.

**Table 2:** Means (standard errors) of standard deviation values for color coordinates measured in triplicate according to teeth and repositioning guides (n = 18)

Color coordinate		L*		a*		b*	
Tooth		Central incisor	Canine	Central incisor	Canine	Central incisor	Canine
Guides	None (freehand)	1.33 (0.24)^Aa^	0.89 (0.13)^Aa^	0.14 (0.02)^Aa^	0.08 (0.02)^Aa^	0.40 (0.07)^Aa^	0.49 (0.06)^Aa^
	Pink silicone	0.57 (0.12)Ba	0.49 (0.17)^Ba^	0.08 (0.01)^Aa^	0.11 (0.03)^Aa^	0.28 (0.06)^Aa^	0.60 (0.24)^Aa^
	Blue silicone	0.58 (0.10)Ba	0.48 (0.08)^Ba^	0.15 (0.03)^Aa^	0.10 (0.02)^Aa^	0.41 (0.06)^Aa^	0.59 (0.24)^Aa^
	Translucent silicone	0.86 (0.24)^Aba^	0.56 (0.09)^Aba^	0.11 (0.02)^Aa^	0.11 (0.03)^Aa^	0.76 (0.39)^Aa^	0.76 (0.27)^Aa^
	Bleaching tray	0.88 (0.18)^ABa^	0.80 (0.11)^ABa^	0.14 (0.02)^Aa^	0.09 (0.02)^Aa^	0.65 (0.30)^Aa^	0.52 (0.06)^Aa^

For each color coordinate, distinct letters (uppercase comparing the repositioning guides, lowercase comparing the teeth) indicate statistical difference at Tukey`s test (p < 0.05).

As for the coordinates a* and b*, neither “tooth” (p = 0.148 and p = 0.250, respectively) nor “repositioning guide” (p = 0.728 and p = 0.400, respectively) had a significant effect on results. Similarly, there were no significant interactions between factors (p = 0.142 for a* and p = 0.312 for b*).

## Discussion

The findings of this study demonstrate that the combination of a repositioning guide and a clinical spectrophotometer frequently produces tooth color discrepancies that surpass the acceptable threshold. Furthermore, our findings indicated that repositioning guides made from colored silicones intensified the color discrepancies in comparison to guides made from more translucent and neutral color materials. Even with the utilization of a very thin and highly translucent bleaching tray, noticeable color discrepancies persisted. Additionally, regarding data variability, repositioning guides demonstrated a tendency to reduce the variations between the measured values for lightness in upper central incisor. Therefore, the hypothesis of this study was also rejected.

The aim of using a repositioning guide is to ensure consistent tooth color measurements of the same area. This method would help to prevent color discrepancies resulting from variations in the placement of the spectrophotometer tip. Repositioning guides are commonly employed in tooth bleaching studies, in which color measurements are taken multiple times throughout the experiment.^13-17^ In our study, we observed no significant differences in data variability for chromatic color coordinates (a* and b*) when comparing the use of a repositioning guide to not using one. However, we did find a positive effect on the measurement of lightness (coordinate L*) in upper central incisors when a repositioning guide was employed. Notably, a reduction in data variability was specifically observed when colored silicones were used as guides. Interestingly, even without a guide, there was minimal data variability between the three color measurements. This can be explained by the fact that the diameter of the spectrophotometer tip (6 mm) covers more than half the length of the clinical crown of upper central incisors (average of 11.3 mm) and upper canines (average of 10.5 mm).^12^ Thus, slight variations in the placement of the spectrophotometer tip within the middle third of these teeth can be compensated for by a larger measuring area.

Despite the widespread use of repositioning guides in clinical trials involving tooth color assessment, there is a lack of studies evaluating their impact on measurement accuracy and reproducibility. A previous study explored this topic by utilizing clear 4-mm-thick sheets designed for mouthguards to construct its guides.^19^ Consistent with our findings, the authors reported that using a repositioning guide improved the reproducibility of lightness measurements. However, no significant effect was observed for the color coordinate b*. Interestingly, they noted that the variation coefficient increased when repositioning guides were used to determine the coordinate a* in upper canines, whereas the opposite trend was observed for freehand measurements.^19^ In our study, we opted to use the standard deviations observed between the three measurements conducted on each tooth instead of relying on the variation coefficient. The issue with using variation coefficients is that the average of the a* values tends to approach zero as positive and negative values are typically observed in tooth color measurements. Consequently, the variation coefficients for this color coordinate are often overestimated, reducing the reliability of comparisons. In another study, the overall color difference between various readings was utilized to assess the reproducibility of the method.^18^ No clinically significant differences were observed in that study when comparing the use of repositioning guides (translucent silicone or bleaching tray) to not using them.^18^

To the best of our knowledge, this is the first study to examine the impact of the color of the silicone used for constructing the guide on potential differences in measured tooth color. Previous studies investigating tooth color with clinical spectrophotometers have utilized various impression materials for creating repositioning guides.^12-17^ However, the specific color of the material used is rarely reported, and there is no standardization regarding the thickness and translucency of guides. As a result, it is possible that the repositioning guide may have some effect on study outcomes, but the true influence of the color measurement method remains unknown. Achieving accurate color measurements relies on multiple factors, including appropriate object illumination. Although the clinical spectrophotometer Easyshade employs the illuminant D65 (daylight), close contact between its tip and the tooth surface is crucial for more precise color determination. However, considering that the tooth surface is not perfectly flat, it is expected that surrounding illumination may have some impact, and the color and opacity of the repositioning guide can influence the illumination conditions of teeth.

Contrary to expectations based solely on surface flatness, the effect of the repositioning guide was more noticeable on the flatter central incisors. This can be attributed to the higher translucency of incisors due to their reduced thickness and lower dentin volume than that of canines. Consequently, the presence of the guide on the palatine surface had a more pronounced impact on the measured tooth color in incisors. When examining each color coordinate individually, using a repositioning guide only affected L* values in central incisors, leading to increased values than those by freehand measurements. This increase in lightness can be explained by the reduced light dispersion in the presence of guides, despite the slight observed difference. Regarding the chromatic coordinates, the presence of a repositioning guide only influenced a* values. Using pink silicone resulted in the tooth color appearing redder (higher value), whereas the opposite was observed for blue and translucent silicones. These results may be attributed to the presence of redder/green backgrounds during color readings or the potential reflection of the silicone color on the buccal tooth surface. Interestingly, although the bleaching tray had no effect on the chromatic coordinates, it still exhibited significant color discrepancies, mainly in the upper incisors, compared to the control.

Using silicone as a repositioning guide offers the advantage of constructing the guide directly in the patient’s mouth, eliminating the need for prior stone working models. However, in our study, all repositioning guides were built over stone models to standardize silicone thickness (approximately 3 mm) and to obtain bleaching trays under the same conditions as the other materials. Colored silicones with a thickness of 3 mm exhibit nearly 100% opacity, and the results observed in our study are likely to be similar even to thicker guides in a clinical setting. On the other hand, the opacity of translucent silicone tends to increase with greater guide thickness, leading to potentially different outcomes. Therefore, the use of a bleaching tray as a repositioning guide yields more reproducible results. However, even though the color discrepancy for the free-hand method was the lowest, using a bleaching tray as a guide resulted in ΔE_00_ values above and slightly below the 50:50% acceptability threshold for color measurement in upper incisors and canines, respectively.^20^ Additionally, the fabrication of a bleaching tray is more time-consuming than that of a silicone guide.

Based on the low data variability observed in freehand measurements and the potential impact of guide color and translucency on measurement precision, it appears that tooth color measurement without any repositioning guide may be the most favorable approach. A limitation of this study is that the effect of repositioning guides on color measurement was determined using the freehand method as the control, which is not affected by guides. Hence, an assumption was made that the correct tooth color was the one measured using the freehand method. However, the accuracy of the Easyshade clinical spectrophotometer, which was used in this study, has been shown to range from 44 to 93%.^24^ Therefore, to establish the true color of a tooth, it is necessary to measure it using a gold standard method, such as a spectroradiometer. Furthermore, inaccurate color determination due to the presence of the repositioning guide may not necessarily impact the results observed in longitudinal studies. If the deviation from true colors remains consistent throughout a study, the difference between two different measurements is unlikely to differ significantly from a more accurate method of tooth color measurement. Nonetheless, further studies are warranted to elucidate these points.

## Conclusions

Using a positioning guide increased the lightness values measured with the spectrophotometer in upper central incisors, whereas no effect was observed in the upper canines. Regardless of the tooth being measured, the spectrophotometer consistently indicated redder colors when a pink silicone guide was utilized, whereas the use of blue or translucent silicones resulted in the opposite effect. In terms of color measurement results, guides made with translucent silicone and a bleaching tray exhibited closer values than those obtained using the freehand method. However, the utilization of a positioning guide failed to significantly reduce the variability of data observed across the various measurements.
